# Urban Youth Perspectives on Food Insecurity during the COVID-19 Pandemic: Evidence from the COACHES Study

**DOI:** 10.3390/nu14030455

**Published:** 2022-01-20

**Authors:** Christine St. Pierre, Win Guan, Jamison Merrill, Jennifer M. Sacheck

**Affiliations:** 1 Milken Institute School of Public Health, The George Washington University, Washington, DC 20052, USA; jsacheck25@gwu.edu; 2 Up2Us Sports, New York, NY 10018, USA; wguan@up2ussports.org (W.G.); jamisonmerrill@gmail.com (J.M.)

**Keywords:** food insecurity, youth food perspectives, dietary quality, COVID-19

## Abstract

School disruptions during the COVID-19 pandemic were a likely threat to food security and exacerbated risk factors associated with poor nutrition and health outcomes among low-income youth. As part of an ongoing school-based study aimed at improving physical activity and dietary behaviors (the COACHES study), associations between youth-reported food insecurity and dietary intake across the pandemic-affected academic year of 2020–2021 were examined. Middle school students (6th and 7th grade, 94% Black/African-American, 92% free-/reduced-price lunch eligible) answered validated surveys on food insecurity and diet and were measured for height and weight for calculation of weight status during Fall 2020 (*n* = 88) and Spring 2021 (*n* = 56). During this time, schools underwent a combination of in-person, hybrid, and remote learning. Nearly half of participants were overweight or obese (47%), and self-reported food insecurity was near 30% at both time points. Less than one-third of youth met fruit and vegetable intake guidelines, and more than half drank two or more sugar-sweetened beverages daily. While controlling for sex, maternal education, and weight status, food insecurity was not significantly associated with fruit and vegetable or sugar-sweetened beverage intake. Independent of weight status, youth were aware of being food insecure, yet it did not have an apparent impact on these food groups of concern. These findings highlight the need for greater understanding of youth perceptions of food insecurity in order to adequately address dietary quality and quantity concerns among children.

## 1. Introduction

School closures during the COVID-19 pandemic disrupted food access for those students who depend on free or subsidized school meals as a major part of their food intake, increasing their risk of experiencing food insecurity. Indeed, the USDA’s annual food security assessment indicated that nearly 15% of households with children experienced food insecurity, defined as lack of access to enough food for an active, healthy life, at some point in 2020 [1]. This was an increase over 2019 and a reversal of the declining trend observed over the previous decade [1].

Food insecurity, primarily a quantitative measure of the amount of food available, can have negative effects on not only dietary quality, but also excess weight gain among youth. The association between food insecurity and lower diet quality is clearer in adults than in children [2], but there is evidence of lower diet quality in children when food insecurity is child-reported [3,4]. Data on youth food security status, however, are typically reported by their caregivers or an adult household representative. Youth perceptions of their own food security status are less widely surveyed. The limited research comparing caregiver- and child-reported food insecurity in the same household has indicated that children may perceive themselves as food insecure, even when their caregivers do not [5,6].

The increased unstructured time related to pandemic-induced school schedule disruptions also raised concerns that risk factors associated with excess weight gain during out-of-school time, including shifts toward lower dietary quality, would be exacerbated [7]. Pre-pandemic studies on changes in youth food consumption patterns during unstructured time already showed lower fruit and vegetable intake [8,9] and increased sugar consumption [9,10,11] relative to school days. In the early months of the pandemic, when home quarantine measures were broadly implemented, studies of children and adolescents found increases in food intake overall and in food groups of concern, such as sugar-sweetened beverages (SSBs) and snack foods [12,13,14]. Youth-reported information became even more difficult to obtain during pandemic-related school closures, particularly among low-income populations, who have historically had disparities in internet access—the primary means for reaching youth when out of school [15].

The aim of the present study was to therefore identify the prevalence of youth-reported food insecurity and to examine the relative link between food insecurity and dietary intake of food groups of concern, namely fruits and vegetables (FV) and SSBs, among low-income, urban youth during the 2020–2021 school year. We further discuss the implications of this evidence for interventions aimed at addressing child food security and dietary quality in disadvantaged communities.

## 2. Materials and Methods

### 2.1. Study Design

Data for this analysis come from the Creating Opportunities for Adolescents through the Coaching, Healthy Eating, and Sports (COACHES) study, which was designed to examine the impact of a sports-based youth development program on middle school students’ physical activity, as well as associated secondary outcomes, including nutrition behaviors and social and emotional well-being. The full COACHES study protocol will be submitted elsewhere. Briefly, five public charter middle schools in New Orleans with comparable demographics (predominantly Black/African American, and >90% qualifying for free or reduced meals) were recruited to a non-randomized, quasi-experimental study implemented over two academic years (Year 1: 2019–2020; Year 2: 2020–2021), with three schools receiving the intervention and two serving as controls. The study was significantly affected by the COVID-19 pandemic, which interfered with the ability to fully implement the intervention. Despite the disruptions to data collection, youth-reported data were obtained at both baseline (October 2020), and follow-up (April/May 2021) from all five schools. The research was approved by the George Washington University Committee on Human Research Institutional Review Board.

### 2.2. Participants

Students in the 6th and 7th grades at all five schools were recruited during the 2020–2021 academic year. Recruitment took place via tabling at in-person school orientations, during which parents/guardians were required to visit the school to fill out forms and pick up electronic equipment for virtual and hybrid learning. COACHES staff introduced the program and obtained parent/guardian consent and student assent for enrollment in the study. Of 784 eligible students across the five schools, 187 (23.9%) enrolled in the study.

Students began the school year in August with a fully virtual learning environment, and a hybrid option was added in October 2020. Throughout the remainder of the school year, learning fluctuated between in-person and virtual, as the city responded to changes in the level of COVID-19 community spread. Furthermore, individual classes could switch to fully virtual learning at any point following a COVID-19 exposure. These schedule fluctuations presented a challenge to arranging in-person data collection, and 86 students (46% of enrolled participants) who were fully virtual had to be excluded due to an inability to collect in-person data. Out of the 101 remaining students, 91 (90.1%) completed surveys in Fall 2020 and 59 (58.4%) completed surveys in Spring 2021. 

### 2.3. Data Collection

Demographic information, including student grade, age, sex, race/ethnicity, free or reduced-price lunch status, and parents’ highest level of education, was collected from the parent/caregiver via a 12-item demographic questionnaire during the study consent process. Data collection from students, including surveys and anthropometric measurements, was conducted in person at each school at each time point. 

A health impact survey was added to the original study protocol and used at both baseline and follow-up to assess the impact of the pandemic on student self-reported mental and physical well-being. The 50-item Coronavirus Health Impact Survey (CRISIS) V0.1 questionnaire [16] was developed for global use across diverse populations and was selected for use in the COACHES study for its holistic assessment of youth physical, mental, and emotional status and daily health-related behaviors. The survey asks respondents to consider the previous two weeks to answer the questions. Of particular interest for the analysis presented here were questions related to food insecurity, sugar-sweetened beverage (SSB), and fruit and vegetable (FV) intake. To assess food security, the survey asked whether the person would “worry about the amount or type of food available to you at home due to money or lack of availability” (yes/no). For food group intake, students were asked to indicate how many fruits and vegetables they ate (0–1, 2–3, 4–5, more than 5) and how many sugar-sweetened beverages they drank (0, 1, 2, 3, 4 or more) on a daily basis. 

Height and weight were measured in private by trained researchers using a portable stadiometer and a digital scale (model 213 and 803, respectively; Seca Weighing and Measuring Systems, Hanover, MD, USA). Measures were taken in triplicate to the nearest 1/8 inch and 0.1 kg and then averaged. Height in inches was converted to meters to calculate body mass index (BMI) (kg/m^2^). Students were classified as underweight (BMI < 5th percentile), healthy weight (5th percentile ≤ BMI < 85th percentile), overweight (85th percentile ≤ BMI < 95th percentile), or obese (BMI ≥ 95th percentile) using the Centers for Disease Control and Prevention age- and sex-appropriate charts [17]. 

### 2.4. Data Analysis

For the purposes of this study, the Fall 2020 and Spring 2021 data were treated as two cross-sectional samples rather than analyzed longitudinally due to lack of comparable pre-pandemic data on these students, a small sample size and challenges to obtaining complete data for individual students due to variations in school operations between virtual, hybrid and in person. Prior to analysis, data were checked for accuracy and missing values. Survey responses with missing answers on the food security question were excluded (*n* = 3 in Fall 2020, *n* = 3 in Spring 2021), bringing the final sample sizes to *n* = 88 in Fall 2020 and *n* = 56 in Spring 2021. For the analysis, FV intake was dichotomized as adequate (4–5 and more than 5 per day) or low (0–3 per day). SSB intake was dichotomized as higher frequency (2 or more per day) or lower frequency (0 and 1 per day). 

Statistical analyses were performed using R (version 3.6.1, R Core Team, Vienna, Austria, 2019). Descriptive statistics were evaluated for each sample. Food insecurity was the primary exposure variable of interest and was tested for a bivariate association with each of the outcomes of interest, high SSB intake and low FV intake, with a chi-square test of independence. Multivariate logistic regression was used to test the association between food insecurity and each respective dietary outcome, controlling for sex, maternal education, and obese weight status. Regression model results were evaluated for statistical significance at the *p* ≤ 0.05 level. 

## 3. Results

### 3.1. Descriptive Statistics

Table 1 presents the demographic characteristics, food insecurity, SSB intake and FV intake for both the Fall 2020 and Spring 2021 samples. The majority of both samples were 7th graders (11.9 ± 0.8 years, range: 10–14 years), female, and predominantly Black/African American. Nearly half were overweight or obese, and none of the students were classified as underweight. More than half of the students came from households where maternal education did not extend beyond high school, and more than 90% of the youth qualified for free or reduced-price meals. There were no statistically significant differences in the characteristics between samples at each time point, nor were there any differences between those assessed twice (*n =* 49) and those only assessed in Fall 2020 (*n* = 39).

More than 25% of students at both time points indicated they had worried about the amount or type of food available to them at home in the two weeks prior to completing the survey. Less than one-third of participants met the recommendation for five fruits and vegetables daily. When responses were analyzed for very low FV intake (0–1 daily), approximately 30% of students fell into this category (28.2% in Fall 2020 and 35.2% in Spring 2021). At both time points, more than half of students were drinking at least two SSBs on a daily basis. When the cut point for SSB intake was lowered to one or more per day, over 80% of respondents at both time points reported drinking SSBs on a daily basis (83.7% in Fall 2020 and 81.8% in Spring 2021). Across all subgroups (e.g., sex, weight status) at both time points, 60% or more of students were not meeting daily fruit and vegetable intake recommendations, and more than 40% of students drank two or more SSBs daily.

### 3.2. Bivariate and Multivariate Analyses

Analysis of the independent associations between food insecurity and FV intake and SSB intake, respectively, did not yield significant results at either time point (Table 2). The proportion of students consuming two or more SSBs daily in the spring was lower for both groups compared with the fall, especially among the food insecure group, but the differences between groups were not sufficient to allow for any conclusions about associations.

Previous studies have indicated potential relationships between food security status, sex, and obesity. To further test for effect modification in our data, we stratified food insecurity by sex and obese weight status given previous studies indicating potential relationships [18,19,20,21,22]. There was no difference in food security status by sex or weight status at either time point (Table 3). Across all subgroups, youth-reported food insecurity remained at or greater than 25% at both time points.

In the logistic regression analyses, there was no significant association between food insecurity and FV or SSB intake when controlling for sex, maternal education, and obese weight status. Odds ratios are presented in Table 4 and Table 5. 

## 4. Discussion

Our findings provide important insights into the food experiences of low-income middle school students during the COVID-19 pandemic, where, to date, there is very limited nutrition-related information available from this population. Youth-reported food insecurity levels were surprisingly high, with nearly a third of students reporting potential food insecurity within the prior two weeks during both the fall of 2020 and spring of 2021. We also observed low FV intake and high SSB intake for the population overall and across subgroups—patterns that were interestingly not affected by food security status. (Unpublished pilot data collected in January 2020 from students at the three COACHES intervention schools showed already low FV intake and high SSB intake pre-pandemic: 50% and 37% were not eating vegetables and fruit, respectively, on a daily basis, and 60% were drinking one or more SSB daily). The lack of association may be due to the consistency of low FV and high SSB intake in this region—our data are consistent with 2019 Youth Risk Behavior Survey results indicating that teenagers in Louisiana had low FV intake and high SSB intake relative to national averages [23]. 

We do not have insight into the pre-pandemic food security situation of these students, but they report a much higher prevalence of food insecurity than the 2020 national average, where child food insecurity was caregiver-reported in 7.6% of households with children [1]. The high prevalence of food insecurity we found at both time points aligns with the limited previous research that indicates an awareness of food insecurity among youth, even when adults in the household try to shield them. Focus groups with youth from low-income areas have indicated that youth experience awareness of food insecurity at cognitive, physical, and emotional levels and engage in strategies such as restricting their own food intake, eating quickly, and helping obtain food for their families in response to perceived food insecurity [24,25]. These findings also exposed differences in the way that adults and children experience food insecurity, and when children and adults from the same families were interviewed separately, caregivers tended to underestimate a child’s awareness of food insecurity in the household [24]. Further work is needed to develop food security assessment tools that are specific to the youth experience, and more studies on child food insecurity should survey children themselves as well as caregivers to ensure the magnitude of child food insecurity is accurately understood. 

Our findings also indicate that food insecurity persisted despite a myriad of interventions during the pandemic aimed at mitigating rising food insecurity, such as community feeding sites for school meals, food delivery directly to students’ homes during virtual learning, and increases in national food assistance programs, some of which provided additional benefits to families with children eligible for free or reduced-price school meals. We are unable to discern from the data which programs were reaching students, but we do know from the five schools that great effort was made to get meals to students through a variety of channels. School meal flexibilities and food assistance expansion at the national level and the work of many on the ground at the local level undoubtedly helped lessen the adverse impacts of the COVID-19 pandemic. Nevertheless, the overlap of food insecurity and dietary quality concerns in the data presented here indicate the importance of addressing diet quality in culturally appropriate ways as a part of the work to ensure food security for all households. This includes increasing the availability of healthful and appealing food options, engaging and empowering youth to make healthy choices through nutrition education, and amplifying community-led efforts to increase the amount of fresh, affordable, healthy foods available in their neighborhoods. 

The high food insecurity, low FV consumption, high SSB consumption, and high prevalence of obesity observed in this low-income, predominantly Black population also has implications for ongoing efforts to prevent and address childhood obesity. Across dozens of studies examining the relationship between food insecurity and obesity among children, there is evidence of a potential differing effect by sex and number of food insecurity experiences, as well as possible variations by race/ethnicity (St. Pierre et al., under review [26]). Since the onset of the COVID-19 pandemic, increases in food insecurity and childhood obesity have had disproportionate effects by race. While food insecurity prevalence decreased among White households from 2019 to 2020, prevalence increased among Black households, and food insecurity among Hispanic households was more than double that of White households [10]. By early 2021, data were showing increasing rates of BMI growth [27] and an increased prevalence of obesity among children and adolescents, which was more pronounced among Black, Hispanic, and lower income youth [28]. The differing implications of food insecurity for childhood obesity and related health outcomes depending on sex and race/ethnicity should further inform efforts to concurrently address food insecurity and dietary quality. 

The present study had several limitations. The challenges to data collection inhibited our ability to follow the same students over time, requiring us to analyze the data cross-sectionally, and our small sample size limits the generalizability of our findings. Second, we do not have information on the food security status of the youth prior to the pandemic and do not have directly comparable pre-pandemic dietary information, so we are unable to discern the effect of the pandemic from economic hardships that may have already existed. Third, there is some risk of bias in our data due to correlation between student responses in the fall and in the spring to the same survey. The specificity of a two-week time frame in the survey and a distance of five months between survey points help mitigate this risk. Fourth, our assessment of dietary quality was limited to SSB and FV consumption, and while these are important food groups for the youth population, they do not give us the entire dietary picture. While more comprehensive assessment tools exist to examine both food security and dietary intake, the limited opportunities for in-person engagement with students required selection of validated instruments that could most efficiently collect information on all of the COACHES study outcomes. These data nevertheless give us important, first-hand insight into the experience of a low-income, urban youth population during the pandemic and can help inform ongoing and future nutrition interventions targeting similar populations. 

## 5. Conclusions

Among this group of middle school students, we observed persistently high SSB intake and low FV intake alongside a high prevalence of youth-reported food insecurity. Future research is needed to gain a deeper understanding of the qualitative aspect of youth-perceived food insecurity to inform interventions to increase food security among children. 

## Figures and Tables

**Table 1:** Descriptive sample characteristics.

Characteristics	Fall 2020 (*n* = 88)	Spring 2021 (*n* = 56)
Grade, %		
6th	42.0	38.5
7th	58.0	61.5
Age (y) ^1^	11.9 ± 0.8	
Female, %	58.9	57.1
Race/Ethnicity, %		
Black/African American	94.3	94.6
Hispanic/Latino	3.4	3.6
Multi-racial	2.3	1.8
Mother’s Education Level, %		
Less than high school	9.0	11.8
High school graduate	47.5	45.1
Some college	20.5	23.5
Higher education degree	20.5	17.6
Don’t know	2.6	2.0
Obese, %	26.0	32.1
Overweight, %	20.8	17.8
Food insecure ^2^, %	28.4	30.3
Low FV intake ^3^, %	68.2	74.1
High SSB intake ^4^, %	68.6	54.5

^1^ Age collected as a whole number at time of consent in Fall 2020, not able to calculate average for spring. ^2^ Over the past 2 weeks; youth-reported. ^3^ Less than four fruits or vegetables daily. ^4^ Two or more sugar-sweetened beverages daily.

**Table 2:** SSB and FV Intake by food security status.

	Fall 2020			Spring 2021		
	Food Secure (*n* = 63)	Food Insecure (*n* = 25)	*p* Value	Food Secure (*n* = 39)	Food Insecure (*n* = 17)	*p* Value
Low FV Intake ^1^	67.2%	70.8%	0.74	76.3%	68.7%	0.69
High SSB Intake ^2^	68.9%	68.0%	0.94	60.5%	41.2%	0.18

^1^ Less than four fruits or vegetables daily. ^2^ Two or more sugar-sweetened beverages daily.

**Table 3:** Food security status by sex and obese weight status.

	Fall 2020			Spring 2021		
	Girls (*n* = 51)	Boys (*n* = 37)	*p* Value	Girls (*n* = 32)	Boys (*n* = 24)	*p* Value
Food Insecure	27.5%	29.7%	0.82	25.0%	37.5%	0.31
	Obese (*n* = 20)	Non-obese (*n* = 57)	*p* Value	Obese (*n* = 18)	Non-obese (*n* = 38)	*p* Value
Food Insecure	25.0%	29.8%	0.68	27.8%	31.6%	0.77

**Table 4:** Odds of low FV intake.

	Fall 2020		Spring 2021	
Variable	Odds Ratio (95% CI)	*p* value	Odds Ratio (95% CI)	*p* value
Sex (female)	0.79 (0.25, 2.50)	0.69	2.76 (0.66, 11.65)	0.17
Lower maternal education	0.67 (0.20, 2.20)	0.51	2.64 (0.65, 10.79)	0.17
Obese weight status	0.68 (0.19, 2.42)	0.56	3.51 (0.63, 19.65)	0.15
Food Insecure	0.88 (0.27, 2.91)	0.83	1.07 (0.24, 4.73)	0.93

**Table 5:** Odds of high SSB intake.

	Fall 2020		Spring 2021	
Variable	Odds Ratio (95% CI)	*p* value	Odds Ratio (95% CI)	*p* value
Sex (female)	2.16 (0.69, 6.78)	0.18	0.62 (0.17, 2.20)	0.46
Lower maternal education	1.17 (0.35, 3.96)	0.79	0.84 (0.24, 2.90)	0.78
Obese weight status	0.55 (0.15, 1.94)	0.35	3.01 (0.77, 11.81)	0.11
Food Insecure	1.41 (0.41, 4.88)	0.58	0.38 (0.10, 1.42)	0.15

## Data Availability

The data presented in this study are available upon request from the corresponding author. The data are not publicly available in accordance with the IRB process regarding confidentiality in participant consent procedures.
